# Fcγ receptor activation mediates vascular inflammation and abdominal aortic aneurysm development

**DOI:** 10.1002/ctm2.463

**Published:** 2021-07-04

**Authors:** Laura Lopez‐Sanz, Susana Bernal, Luna Jimenez‐Castilla, Ignacio Prieto, Sara La Manna, Sergio Gomez‐Lopez, Luis Miguel Blanco‐Colio, Jesus Egido, Jose Luis Martin‐Ventura, Carmen Gomez‐Guerrero

**Affiliations:** ^1^ Renal, Vascular and Diabetes Research Lab IIS‐Fundacion Jimenez Diaz (IIS‐FJD) Madrid Spain; ^2^ Universidad Autonoma de Madrid (UAM) Madrid Spain; ^3^ Spanish Biomedical Research Centre in Diabetes and Associated Metabolic Disorders (CIBERDEM) Madrid Spain; ^4^ Spanish Biomedical Research Centre in Cardiovascular Diseases (CIBERCV) Madrid Spain

**Keywords:** abdominal aortic aneurysm, antibodies, autoantigens, Fc receptors, immune system, inflammation, oxidative stress

## Abstract

**Background:**

Abdominal aortic aneurysm (AAA), a degenerative vascular pathology characterized by permanent dilation of the aorta, is considered a chronic inflammatory disease involving innate/adaptive immunity. However, the functional role of antibody‐dependent immune response against antigens present in the damaged vessel remains unresolved. We hypothesized that engagement of immunoglobulin G (IgG) Fc receptors (FcγR) by immune complexes (IC) in the aortic wall contributes to AAA development. We therefore evaluated FcγR expression in AAA lesions and analysed whether inhibition of FcγR signaling molecules (γ‐chain and Syk kinase) influences AAA formation in mice.

**Methods:**

FcγR gene/protein expression was assessed in human and mouse AAA tissues. Experimental AAA was induced by aortic elastase perfusion in wild‐type (WT) mice and γ‐chain knockout (γKO) mice (devoid of activating FcγR) in combination with macrophage adoptive transfer or Syk inhibitor treatment. To verify the mechanisms of FcγR in vitro, vascular smooth muscle cells (VSMC) and macrophages were stimulated with IgG IC.

**Results:**

FcγR overexpression was detected in adventitia and media layers of human and mouse AAA. Elastase‐perfused γKO mice exhibited a decrease in AAA incidence, aortic dilation, elastin degradation, and VSMC loss. This was associated with (1) reduced infiltrating leukocytes and immune deposits in AAA lesions, (2) inflammatory genes and metalloproteinases downregulation, (3) redox balance restoration, and (4) converse phenotype of anti‐inflammatory macrophage M2 and contractile VSMC. Adoptive transfer of FcγR‐expressing macrophages aggravated aneurysm in γKO mice. In vitro, FcγR deficiency attenuated inflammatory gene expression, oxidative stress, and phenotypic switch triggered by IC. Additionally, Syk inhibition prevented IC‐mediated cell responses, reduced inflammation, and mitigated AAA formation.

**Conclusion:**

Our findings provide insight into the role and mechanisms mediating IgG‐FcγR‐associated inflammation and aortic wall injury in AAA, which might represent therapeutic targets against AAA disease.

## Graphical abstract

Our work suggests a pathogenic role of IgG IC interaction with activating FcγR present in infiltrating and VSMC during AAA formation. These results support the investigation of novel immunotherapeutic strategies targeting FcγRs and/or downstream molecules to inhibit humoral immune damage in AAA

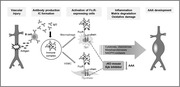

**Abbreviations**
8OHdG: 8‐hydroxy‐2′‐deoxyguanosine
AAA: abdominal aortic aneurysm
ACTA2: actin‐α2
ARG: Arginase
BM: bone marrow
CCL: C‐C motif ligand
CD206: macrophage mannose receptor‐1
CXCL10: C‐X‐C motif chemokine 10
DHE: dihydroethidium
DMEM: Dulbecco´s Modified Eagle Medium
FBS: foetal bovine serum
FcγR: immunoglobulin G Fc receptor
IC: immune complex
ICAM1: intercellular adhesion molecule‐1
IFNγ: interferon‐γ
Ig: immunoglobulin
IL: interleukin
iNOS: inducible nitric oxide synthase
ITAM: immunoreceptor tyrosine‐based activation motif
ITIM: immunoreceptor tyrosine‐based inhibition motif
KLF‐4: Krueppel‐like factor‐4
MMP: metalloproteinase
NOX: nicotine adenine dinucleotide phosphate oxidase
SM22α: smooth muscle protein 22‐α
SOD1: superoxide dismutase 1
TIMP: tissue inhibitor of metalloproteinase
TNFα: tumour necrosis factor‐α
VSMC: vascular smooth muscle cells
VVG: Verhoeff‐van Gieson
WT: wild‐type
α‐SMA: α‐smooth muscle actin
γKO: γ‐chain knockout

## 1 INTRODUCTION

Abdominal aortic aneurysm (AAA) is a multifactorial degenerative disease of the aortic wall characterized by progressive weakening and dilation of the abdominal aorta. AAA occurs mainly in adults and causes 1.3% of deaths in men of 65‐85 years of age in developed countries. Many patients with AAA are asymptomatic and their aneurysmal lesions are detected incidentally on diagnostic imaging. The average growth rate of small AAA is 0.3‐0.5 cm/year; when the aneurysm is symptomatic and/or larger than 5.5 cm in diameter, the surgical repair is indicated to prevent a life‐threatening aortic dissection or rupture.^1^ Besides conventional treatment including lipid‐lowering drugs, antihypertensives and antiplatelet drugs, new pharmacological therapies to reduce the growth rate of AAA or to prevent its rupture are not sufficiently developed.^2^ This is, in part, because the pathogenic mechanisms contributing to AAA formation and progression are not yet understood. Pathologic features of AAA include progressive destruction of the elastic media layer, vascular smooth muscle cell (VSMC) dysfunction, adventitial and medial inflammatory cell infiltration, and enhanced oxidative stress in the vessel wall.^3^

Nowadays, AAA is considered a chronic inflammatory disease with a strong innate and adaptive immune component.^4^ In AAA lesions, infiltrating immune cells (macrophages, neutrophils, mast cells, B, and T lymphocytes) along with VSMC and fibroblasts, produce cytokines, and matrix metalloproteinases (MMP) that can locally promote an inflammatory reaction, extracellular matrix degradation, VSMC apoptosis, phenotypic switch and neovascularization, further weakening the vessel wall and making it susceptible to rupture.^3^, ^5^

In contrast to the well‐characterized function of cell‐mediated immunity, the role of antibody‐dependent adaptive immunity in AAA formation is less understood. Studies have detected B cells and their antibodies (predominantly immunoglobulin G [IgG] but also IgM and IgE isotypes) in AAA tissues, mainly distributed in organized follicle‐like structures.^6^, ^7^ IgG antibodies from human aneurysmal tissues are immunoreactive with aortic wall components.^8^, ^9^ Circulating and/or tissue levels of IgG against autoantigens (eg, phospholipids, lipoproteins and lipid peroxidation products) are associated with AAA progression.^10^, ^11^, ^12^ In rodents, circulating IgG recognizing specific epitopes in AAA tissues can activate inflammation and complement system,^13^, ^14^, ^15^, ^16^ but the mechanisms and effector molecules remain ambiguous.

Aside from their ability to bind to antigens, IgG antibodies and immune complexes (IC) influence inflammation by interacting with receptors specific for the IgG Fc constant region (FcγR) on the cell surface of infiltrating and resident cells. The human IgG receptor family consist of six classical FcγR (FcγRIA/CD64A, FcγRIIA/CD32A, FcγRIIB/CD32B, FcγRIIC/CD32C, FcγRIIIA/CD16A, and FcγRIIIB/CD16B), complemented by FcR‐like receptors homologous to FcγRIA (hFcRL4/CD307d and hFcRL5/CD307e), and FcRn and TRIM21 involved in recycling and transport of IgG.^17^ The FcγR family members differ in affinity and specificity for the different IgG forms, signaling pathways, cellular distribution, and effector functions. In mice, four different classes have been described: the high‐affinity FcγRI/CD64 involved in monomeric IgG binding, and the low‐/medium‐affinity FcγRIIB/CD32B, FcγRIII/CD16, and FcγRIV/CD16‐2, which interact with multivalent IgG.^18^ Human and mouse FcγRI/CD64 and FcγRIIB/CD32B are orthologs, human FcγRIIA/CD32A is the equivalent of mouse FcγRIII/CD16, and human FcγRIIIA/CD16A is similar to mouse FcγRIV/CD16‐2, whereas FcγRIIC/CD32C and FcγIIIB/CD16B are not present in mice (Supplementary Figure S1A). There are also reported high/low responder polymorphisms for FcγIIA (R131H/R) and FcγRIIIA (F158F/V) affecting ligand binding and effector functions.^17^ Activating FcγR isotypes (human IA and IIIA; mouse I, III, and IV) associate with γ‐chain, the common subunit carrying the immunoreceptor tyrosine‐based activation motif (ITAM) required for receptor assembly and signal transduction by sequential activation of Src and Syk tyrosine kinases.^19^ Counterbalancing activating FcγR, the inhibitory receptor FcγRIIB contains the immunoreceptor tyrosine‐based inhibition motif (ITIM) that is phosphorylated and recruits the inositol 5‐phosphatase to negatively regulate innate and adaptive immunity.^17^, ^18^

By analyzing the phenotype of mice deficient in either individual FcγR or the common γ‐chain, we and others have shown that activating FcγR contribute to atherosclerotic lesion formation,^20^, ^21^, ^22^ while FcγRIIB has a controversial (both protective and pathogenic) role.^23^, ^24^ In the AAA setting, few studies so far have investigated FcγRIIB expression^25^, ^26^ and Syk activation^27^ in human AAA; however, the function and cell types expressing FcγR have not been scrutinized. Therefore, the aim of this study is to explore the contribution of FcγR‐mediated responses to inflammation and tissue injury during AAA formation. We examined FcγR isotypes expression in human AAA samples. The functional role of activating FcγR and Syk was further explored in the elastase‐perfused mice, a nondissecting AAA model with pathological similitudes to human lesions such as degradation of elastin fibers in the media and inflammatory cell accumulation in the adventitia.^2^ In vivo studies were encompassed by mechanism experiments in cells stimulated with IgG IC.

**HIGHLIGHTS**

- Human and mouse AAA lesions show increased expression of FcγR isoforms in adventitia and media layers.
- Blockade of activating FcγR signaling by γ‐chain gene deficiency or Syk kinase inhibition limits AAA formation in elastase‐perfused mice.
- FcγR inhibition in VSMC and macrophages downregulates inflammatory genes and metalloproteinase activity and alters redox balance and phenotypic switch.

## 2 METHODS

### 2.1 Experimental mouse model of AAA

Mice carrying a single genetic deficiency in the FcγR common γ‐chain^28^, ^29^ (γKO) and wild‐type (WT) littermates (*Mus musculus*, C57BL/6J, males, 10‐12 weeks old, 23‐28 g body weight) were bred and maintained at the Animal Facility of IIS‐Fundacion Jimenez Diaz. Mice were housed in ventilated cages (2‐4 animals/cage) in a temperature‐controlled room (20‐22°C and 12‐hour light/dark cycle) with environmental enrichment and bedding, and free access to water and standard food. Studies were conducted under 3R principle (replacement/refinement/reduction) in accordance with Directive 2010/63/EU of European Parliament and were approved by the Institutional Animal Care and Use Committee and Comunidad de Madrid (PROEX 217/19).

Experimental AAA was induced by aortic perfusion of elastase from porcine pancreas (type I, specific activity 7 units/mg protein; E1250, Sigma‐Aldrich) in WT and γKO mice, as previously described.^30^, ^31^ In brief, mice were anaesthetized by 2% isoflurane inhalation and underwent laparotomy. The infrarenal abdominal aorta was isolated with the assistance of a surgical stereomicroscope and temporarily ligated between the renal and iliac arteries. An aortotomy was performed using a 30‐gauge needle, the aorta was exsanguinated, and a PE‐26 polyethylene tubing was introduced through the aortotomy. Then, the aorta was infused with elastase (1:2 dilution in saline solution) for 5 minutes at 100 mmHg. The Sham group received saline instead of elastase. After infusion, the aortotomy was repaired, ligation removed, aortic flow restored, and incision closed. Animals were housed under standard conditions. On day 14 postsurgery, mice were euthanized under anesthesia (ketamine 100 mg/kg and xylazine 10 mg/kg), and blood and aorta samples were collected.

Type and number of samples: abdominal aortas for histology (WT‐Sham, *n* = 8; WT‐Elastase, *n* = 12; γKO‐Sham, *n* = 7; γKO‐Elastase, *n* = 12); abdominal and thoracic aortas for mRNA expression (WT‐Sham, *n* = 8; WT‐Elastase, *n* = 10; γKO‐Sham, *n* = 7; γKO‐Elastase, *n* = 8); abdominal aortas for protein expression (WT‐Elastase, *n* = 9; γKO‐Elastase, *n* = 9); sera for zymography (WT‐Sham, *n* = 5; WT‐Elastase, *n* = 9; γKO‐Sham, *n* = 5; γKO‐Elastase, *n* = 9).

### 2.2 Adoptive transfer and Syk inhibition in vivo

Donor mice (WT and γKO) anesthetized with 2% isoflurane were sacrificed by cervical dislocation. Bone marrow (BM) cells were collected from femurs and tibias and cultured in Dulbecco´s Modified Eagle Medium (DMEM; D6546, Sigma‐Aldrich) containing 10% fetal bovine serum (FBS) and 10% L929 cell‐conditioned medium as a source of macrophage colony stimulating factor.^21^ After 6‐7 days, adherent BM‐derived macrophages were gently detached from plates and resuspended to 1 × 10^7^ cells/mL. Two‐hundred microliters were injected intravenously via the tail vein in recipient mice (WT and γKO; *n* = 5 each group) the day before elastase infusion and every 2‐3 days thereafter for the following 14 days. In pilot experiments, macrophages labeled with 4 mM 5‐chloromethylfluorescein diacetate (C7025, Invitrogen) were transferred into mice at 7th day of elastase‐AAA induction. After 24‐48 hours, distribution was examined by ex vivo imaging (IVIS Lumina system; Caliper Life Sciences) in longitudinal aorta, fluorescence microscopy in aortic cross‐sections, and peripheral blood flow cytometry with anti‐CD11b antibody (BD Biosciences Cat# 552850, RRID:AB 394491). This cell labeling and tracking method has limitations by biological effects (eg, secondary phagocytosis of dead cells or released fluorescent probe by host cells), but it helped to further design our adoptive transfer strategy.

For in vivo inhibition of Syk kinase, WT mice were treated with Bay 61‐3606 (S7006, Selleckchem; 50 mg/kg body weight, intraperitoneally, 3 days per week). Dose and administration route were chosen based on the previous literature.^32^ For preventive treatment, mice (*n* = 7) received Bay 61‐3606 for a period of 2 weeks (from day −1 until day 14 postperfusion). For therapeutic treatment, mice (*n* = 5) received Bay 61‐3606 for 1 week (from day 7 until day 14 postperfusion). Mice treated with vehicle (0.1% DMSO, *n* = 7) and Sham control (*n* = 6) were also included. Aorta sampling and histology were carried out at day 14.

### 2.3 Histology and immunohistochemistry

At the end of the experimental models, mouse aortas were harvested and cleaned, then fixed in 10% neutral buffered formalin for paraffin embedding. Aortic wall thickness (medial area) and perimeter (luminal circumference) were measured in interval 4‐μm cross‐sections after Masson's trichrome staining. In each mouse, sections with the maximum size were used to calculate the aortic luminal diameter from the perimeter. The percentage of aortic diameter increase was calculated from the difference between preperfusion and final values, and AAA was defined as ≥100% increase. The severity of AAA was further evaluated in serial cross‐sections of abdominal aorta stained with Verhoeff‐van Gieson (VVG) procedure to observe the integrity of elastin fibers, and with α‐smooth muscle actin antibody (α‐SMA Cy3 conjugated; Sigma‐Aldrich Cat# C6198, RRID:AB_476856) with 4′,6‐diamidino‐2‐phenylindole (DAPI) nuclear counterstain to detect VSMC content. Medial elastin integrity was scored by two observers as follows: grade 1, intact, well‐organized elastic laminae; grade 2, elastic laminae with some disruptions; grade 3, multiple disruptions; and grade 4, severe elastin fragmentation or loss. VSMC content in the tunica media was scored by two observers as follows: grade 1, intact VSMC; grade 2, minimal abnormalities; grade 3, loss of few VSMC; and grade 4, severe loss of VSMC in the media.^30^, ^31^

For immunohistochemical analysis, following antigen retrieval (0.01 M citrate pH 6, 96°C, 20 minutes), blockade of endogenous peroxidase (3% H_2_O_2_ in methanol, 30 minutes) and nonspecific binding (6% host serum, 30 minutes), aortic sections were incubated overnight at 4°C with primary antibodies against mouse FcγRI/CD64 (R&D Systems Cat# AF2074, RRID:AB_416550), FcγRIII/CD16 (R&D Systems Cat# AF1960, RRID:AB_355077), FcγRIIB/CD32B (Cell Signaling Technology Cat# 96397, RRID:AB_2800262), IgG (Jackson ImmunoResearch Labs Cat# 715‐035‐150, RRID:AB_2340770), IgM (Sigma‐Aldrich Cat# A8786, RRID:AB_258413), CD68 (Abcam Cat# ab53444, RRID:AB_869007), Ly6G (BioLegend Cat# 108402, RRID:AB_313367), CD45R (BD Biosciences Cat# 550286, RRID:AB_393581), CD3 (Agilent Cat# A0452, RRID:AB_2335677), S100A4 (Sigma‐Aldrich Cat# HPA007973, RRID:AB_1079858), 8‐hydroxy‐2′‐deoxyguanosine (8OHdG; Abcam Cat# ab10802, RRID:AB_297482), intercellular adhesion molecule‐1 (ICAM1, Abcam Cat# ab124760), phosphorylated Syk (p‐Syk; Tyr323, Tyr317; Thermo Fisher Scientific Cat# 44‐234G, RRID:AB_2533612), and complement C3 and C9 (kindly provided by Dr. Rodriguez de Cordoba, CSIC, Madrid). Samples were rinsed in PBS and sequentially treated with biotinylated secondary antibodies, avidin‐biotin complex (Vector Laboratories), 3, 3‐diaminobenzidine or 3‐amino‐9‐ethylcarbazole (DAKO) peroxidase substrate, and hematoxylin counterstain. Isotype‐matched antibodies were included as negative controls. Superoxide anion was detected by fluorescence microscopy using the redox‐sensitive probe dihydroethidium (DHE 2 μM; Molecular Probes).^31^ Histopathological evaluations were conducted in a blinded manner. Positive staining was quantified as percentage of total area in at least 2 sections per mouse using Image‐Pro Plus software (Media Cybernetics).

### 2.4 Human AAA samples

Aortic wall specimens were obtained during surgical repair from AAA patients (*n* = 10) enrolled in the RESAA (REflet Sanguin de l'evolutivite des Anevrysmes de l'Aorte abdominale) protocol.^33^ Healthy abdominal aortas (*n* = 10) were sampled from brain‐deceased organ donors. The aortic tissues were washed and preserved at 4°C in Ringer´s lactate solution, then dissected into media and adventitial layers and kept at −80°C until further processing for RNA expression analysis. Patient informed consent and ethical committee advice were obtained (RESAA and AMETHYST studies, CPP Paris‐Cochin n° 2095, 1930 and 1931, Inserm Institutional Review Board, IRB0000388). Healthy human aortas were obtained with the authorization of the French Biomedicine Agency (PFS 09‐007, BBMRI network, BB‐0033‐00029). All human studies conformed to the principles outlined in the Declaration of Helsinki.

Immunohistochemistry was done on paraffin‐embedded sections of AAA tissue (4 μm) using antibodies against human FcγRIA/CD64A (Bioss Cat# bs‐3511R, RRID:AB_10856930), FcγRIIIA/CD16A (Bioss Cat# bs‐6028R, RRID:AB_11065672), IgG (H+L) (Sigma‐Aldrich Cat# SAB3701329), CD68 (Agilent Cat# GA60961‐2, RRID:AB_2661840), and α‐SMA (Abcam Cat# ab5694, RRID:AB_2223021) followed by peroxidase‐linked secondary antibodies. For colocalization of FcγR with macrophages and VSMC, double immunofluorescence was performed. Samples were simultaneously incubated with primary antibodies (anti‐FcγRIA/CD64A or anti‐FcγRIIIA/CD16A and anti‐CD68) followed by secondary antibodies (donkey antirabbit IgG, Alexa Fluor 488; Thermo Fisher Scientific Cat# A‐21206, RIDD: AB 2535792; goat‐antimouse IgG, Alexa Fluor 568; Molecular Probes Cat# A‐11004, RRID:AB_2534072) or anti‐αSMA IgG Cy3. Samples were mounted with antifade Vectashield medium with DAPI (Vector Lab) and images obtained using an Axioscope microscope (Carl Zeiss).

### 2.5 Cell cultures

VSMC were isolated from WT and γKO mouse aorta by enzymatic digestion (collagenase type II; C6885, Sigma‐Aldrich), then cultured in DMEM containing 10% FBS, 100 U/mL penicillin, 100 μg/mL streptomycin, and 2 mM L‐glutamine (Sigma‐Aldrich), and used between 2nd and 8th passages as previously reported.^20^ Murine BM‐derived macrophages were cultured for 6‐7 days in DMEM containing 10% FBS and 10% L929 cell‐conditioned medium and freshly used without passaging. VSMC and macrophages were made quiescent (DMEM with 0.5% FBS, 24 hours) and incubated for additional 24 hours with fibrinogen alone (10 μg/mL), fibrinogen‐IgG IC, or heat‐aggregated IgG (150 μg/mL). The IC were prepared by incubating native fibrinogen (10 μg/mL in PBS; Merck Cat# 341578) with rabbit IgG against human fibrinogen (50 μg/mL in PBS; MyBioSource Cat# MBS592295) for 60 minutes at 37°C and 48 hours at 4°C, then filter‐sterilized and stored at –80°C before use. Mouse IgG (Thermo Fisher Scientific Cat# ICN55939, RRID:AB_2334714) was aggregated at 63°C for 30 minutes. For Syk inhibition, cells were treated with Bay 61‐3606 (0.1‐5 μM) for 60 minutes before IC stimulation. In some experiments, MB‐derived macrophages were exposed to lipopolysaccharide (LPS, 100 ng/mL; Sigma‐Aldrich) plus interferon‐γ (IFNγ, 20 ng/mL; PeproTech) or interleukin (IL)‐4 (20 ng/mL; PeproTech) for M1 or M2 polarization, respectively. Cell viability was assessed by the 3‐(4,5‐dimethylthiazol‐2‐yl)‐2,5‐diphenyltetrazolium bromide tetrazolium assay, using medium with 10% FBS and 10% DMSO as positive and negative controls, respectively.

### 2.6 Real‐time PCR analysis

Human AAA and healthy wall tissues were snap frozen in liquid N_2_ and homogenates (0.2 g) were divided and resuspended for mRNA analysis. Total mRNA from human/mouse aorta and cultured cells was extracted with TRI Reagent (Molecular Research Center, Thermo Fisher Scientific). Complementary DNA was synthesized by reverse transcription (High‐Capacity cDNA RT Kit, Applied Biosystems). Quantitative real‐time PCR was performed on a TaqMan ABI 7700 system using Taqman or SYBR Green probes for amplification of the following genes: a) human *FCGR1A*, *FCGR2A*, *FCGR2B*, *FCGR3A*, and *FCGR3B*; b) mouse *Fcgr1*, *Fcgr2b*, *Fcgr3*, Fcgr4, C‐C motif ligand‐2 (*Ccl2*), *Ccl5*, C‐X‐C motif chemokine 10 (*Cxcl10*), *Ifnγ*, tumor necrosis factor‐α (*Tnfα*), *Il‐10*, *Il‐17*, *Icam1*, inducible nitric oxide synthase (*Inos*), arginase 1 and 2 (*Arg1* and *Arg2*), macrophage mannose receptor 1 (*Cd206*), Krüppel‐like factor 4 (*Klf4*), actin‐α2 (*Acta2*), smooth muscle protein 22α (*Sm22α*), *Mmp2*, *Mmp9*, tissue inhibitor of metalloproteinase 1 and 2 (*Timp1* and *Timp2*), nicotine adenine dinucleotide phosphate oxidase subunits (*Nox1*, *Nox2*, *Nox4*, *p47phox* and *p67phox*), catalase (Cat), and superoxide dismutase 1 (*Sod1*). For each sample, expression data were normalized to either human GAPDH or mouse 18S rRNA housekeeping expression. Values were expressed in arbitrary units or converted to fold increases versus Sham mice or cell basal conditions.

### 2.7 Analysis of protein expression

VSMC and macrophages were lysed (10 mM Tris‐HCl pH 7.4 buffer, 150 mM NaCl, 0.5% Nonidet P40, 1% Triton X‐100, 1 mM EDTA, 1 mM EGTA, 0.2 mM Na_3_VO_4_, 10 mM NaF, and protease inhibitors) and total proteins (40 μg) were electrophoresed and immunoblotted for p‐Syk (Thermo Fisher Scientific Cat# 44‐234G, RRID:AB_2533612) and β‐actin (Santa Cruz Biotechnology Cat# sc‐47778 HRP, RRID:AB_626632). Densitometry values normalized to loading control were expressed in arbitrary units or converted to fold increases versus cell basal conditions. Concentrations of secreted proteins (CCL2, CCL5, and TNFα) in cell supernatants were measured in duplicate by ELISA (R&D Systems and Thermo Fisher Scientific).

### 2.8 Gelatin zymography for MMPs

Detection of MMP activity was done by gelatin zymography method. Cell culture supernatants were centrifuged for removing debris and 10 times concentrated (Microcon‐10 kDa, Millipore). Mouse sera were diluted 10‐20 times. Equal amounts of protein samples were run on 10% Zymogram Plus Gelatin Protein Gels (ZY00100BOX; Thermo Fisher Scientific) with 25 mM Tris buffer pH 8.3 containing 250 mM glycine and 0.1% SDS. After electrophoresis, gels were rinsed thrice for 30 minutes with washing buffer containing 2.5% Triton X‐100 and 30 minutes with H_2_O, then incubated overnight at 37°C in 50 mM Tris‐HCl pH 7.5 containing 200 mM NaCl and 100 mM CaCl_2_. Gels were stained for 30 minutes with 0.5% Coomassie brilliant blue R‐250 in 20% methanol and 10% acetic acid. Clear bands corresponding to areas of gelatinase activity were visualized against a blue background. Densitometry of active MMP2/9 bands was expressed as arbitrary units or fold changes versus basal conditions.

### 2.9 Statistics

The result values are presented as individual data and mean ± SD from separate experiments and subjects. In these analyses, technical replicates were averaged to give a single value per biological condition. Statistical analysis was done using GraphPad Prism v.8 (GraphPad Software). Values passed the Pearson's omnibus and D'Agostino normality test and the Barlett test for homogeneity of variance. Statistical significance was established at *P *< 0.05 with unpaired two‐tailed Student's *t*‐test or one‐way ANOVA plus Bonferroni multiple comparison test. Values expressed as fold changes versus Sham group or basal condition to avoid unwanted source of variation were analyzed by nonparametric Mann‐Whitney or Kruskal‐Wallis with Dunn's comparison test.

### 2.10 Data availability

The data supporting the findings of this study are available from the corresponding author upon reasonable request.

## 3 RESULTS

### 3.1 Expression of FcγR in human and experimental AAA

**FIGURE 1:**
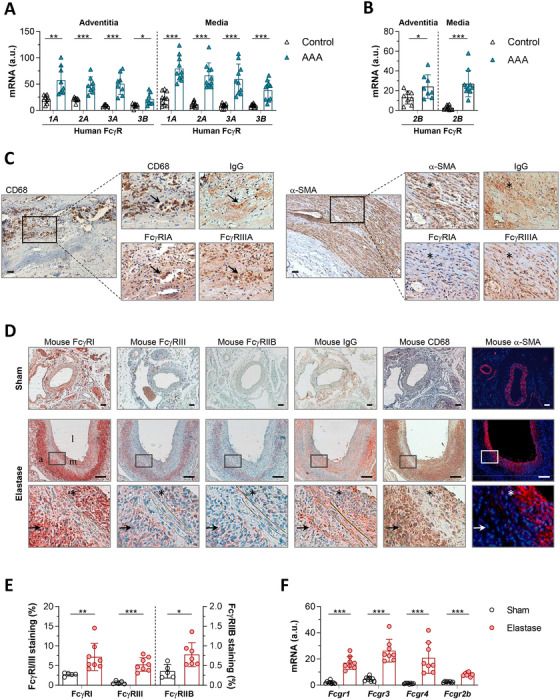
Expression of FcγR subtypes in human and experimental AAA. Quantitative real‐time PCR of human activating FcγR (IA, IIA, IIIA, and IIIB) (A) and inhibitory FcγRIIB (B) in aortic samples from controls and AAA patients. The adventitia (Control, *n* = 10; AAA, *n* = 10) and media (Control, *n* = 8; AAA, *n* = 10) layers were analyzed separately. (C) Representative images (scale bar, 50 μm) and high‐magnification fields (rectangular areas) in human AAA samples showing immunodetection of CD68^+^ macrophages and α‐SMA^+^ VSMC and colocalization with IgG, FcγRIA, and FcγRIIIA. Arrows and asterisks represent macrophage‐ and VSMC‐rich areas, respectively. (D) Representative images (scale bar, 50 μm; l, lumen; m, media; a, adventitia) and high‐magnification fields (rectangular areas) of mouse FcγR (activating I and III; inhibitory IIB), IgG, CD68, and α‐SMA immunostaining in serial aortic cross‐sections from saline‐perfused controls (Sham) and elastase‐perfused mice. Arrows and asterisks denote macrophage and VSMC‐stained areas, respectively. (E) Quantification of FcγR positive staining in mouse groups (Sham, *n* = 5; Elastase, *n* = 7). (F) Real‐time PCR analysis of mouse FcγR expression in aortic samples from Sham and Elastase groups (*n* = 8 mice/group). PCR values normalized to human GAPDH or mouse 18S rRNA are expressed as arbitrary units (a.u.). Results are presented as individual data points and mean ± SD of the total number of samples per group. *P < 0.05, **P < 0.01, and ***P < 0.001 (two‐tailed Student's *t*‐test)

To investigate the involvement of FcγR in human AAA, real‐time PCR analysis was performed in aortic samples from AAA patients and controls. Results demonstrated significant increases in the gene expression of activating FcγR (IA, IIA, IIIA, and IIIB; Figure 1A) and inhibitory FcγRIIB (Figure 1B) in the adventitia and media layers of AAA samples when compared to healthy aortas. Furthermore, immunoperoxidase and double immunofluorescence in AAA tissue sections revealed FcγRIA and FcγRIIIA protein expression colocalizing with IgG, CD68^+^ macrophages, and α‐SMA^+^ VSMC‐stained areas (Figures 1C, Supplementary Figure S1B‐E).

These human findings were further verified in the mouse AAA model. As compared with Sham operated animals, aortic samples from elastase‐infused mice exhibited an upregulation of FcγR isoforms (activating I and III, inhibitory IIB) at both protein and mRNA levels (Figure 1D‐F). In addition, FcγR‐expressing cells colocalized with IgG, CD68, and α‐SMA‐stained areas in AAA lesions (Figure 1D).

### 3.2 Functional deficiency in activating FcγR protects mice against elastase‐induced AAA

**FIGURE 2:**
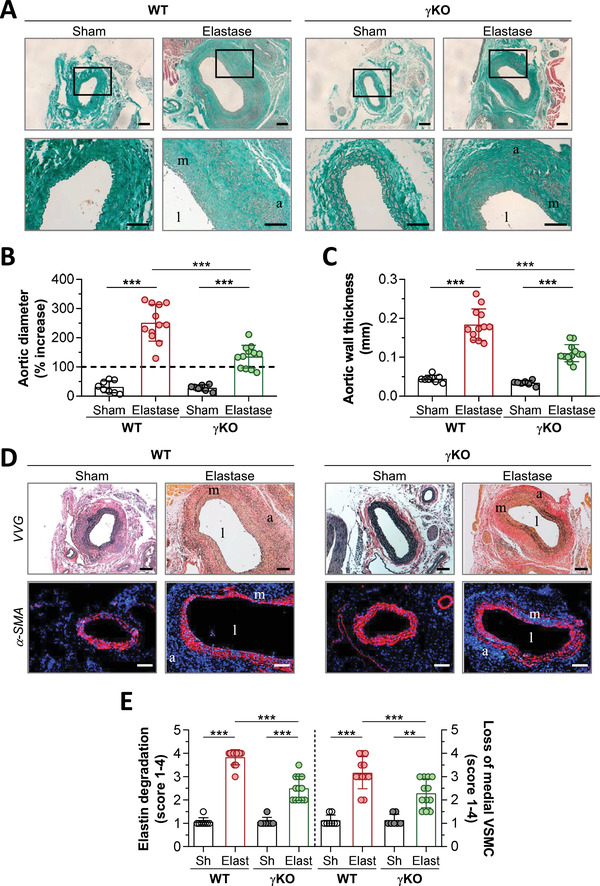
Activating FcγR deficiency reduces elastase‐induced AAA formation in mice. (A) Representative images (scale bars, 100 μm) and high‐magnification fields (rectangular areas) of Masson's trichrome staining in abdominal aortic sections from WT and γKO mice at 14 days postperfusion with either saline or elastase. Quantifications of the aortic diameter increase (B) and the wall thickness (C) in Masson‐stained sections. (D) Representative images (scale bars, 100 μm) of VVG and α‐SMA staining. (E) Quantification of elastin degradation and medial VSMC loss. Results are presented as individual data points and mean ± SD of the mouse groups (WT‐Sham, *n* = 8; WT‐Elastase, *n* = 12; γKO‐Sham, *n* = 7; and γKO‐Elastase, *n* = 12). * * **P < 0.01 and ***P < 0.001 (one‐way ANOVA plus Bonferroni test). l, lumen; m, media; a, adventitia

To further analyze the impact of FcγR on AAA formation, elastase perfusion AAA model was performed in γKO mice that lack the common γ‐chain subunit necessary for signaling and expression of activating FcγR isotypes (I, III, and IV). At day 14 after AAA induction, exploration of abdominal aortic segments from elastase‐infused mice (WT and γKO groups) revealed a higher lumen of aorta compared with their respective Sham controls (Figure 2A). However, the incidence of aneurysm formation was higher in WT group (100%, 12 of 12) than in γKO group (67%, 8 of 12) (Figure 2B). AAA lesions in γKO mice showed a marked and significant decrease of maximal aortic diameter and wall thickness (54% ± 4% and 60% ± 3% of decrease, respectively; *P *< 0.0001) (Figure 2B, C), lesser degrees of elastin degradation (VVG staining) and improved preservation of medial VSMC content (α‐SMA immunofluorescence) (Figure 2D, E).

Because FcγR colocalized with CD68^+^ macrophages in human and mouse aneurysm, we further analyzed the specific role of monocyte/macrophage FcγR activity in AAA. To do this, we prepared BM‐derived macrophages from WT and γKO mice and adoptively transferred them intravenously into either WT or γKO recipient mice (Supplementary Figure S2A). Adoptive transfer of fluorescence‐labeled macrophages showed distribution in longitudinal and cross‐sections of aorta within 24‐48 hours, constituting about 50% of the total CD11b^+^ population in peripheral blood at 24 hours (Supplementary Figure S2B‐D). Elastase‐perfused WT mice receiving γKO macrophages exhibited a partial reduction in AAA (% aortic diameter: WT → WT, 243±11; γKO → WT, 186±7, *P *= 0.0024) and lesser degrees of medial elastin degradation (34% decrease vs WT → WT). Conversely, WT macrophage transfer into γKO recipient mice exacerbated AAA, as evidenced by increased aortic dilation (% aortic diameter: γKO → γKO, 112±7; WT → γKO, 149±5; *P* = 0.0034) and a 26% higher degree of elastin degradation (Supplementary Figure S2E‐I). These data suggest that macrophage FcγR activation plays a central role on AAA formation, although other cell types may also contribute to the pathologic process.

### 3.3 Lack of activating FcγR attenuates inflammation and immune response in AAA lesions and cultured cells

**FIGURE 3:**
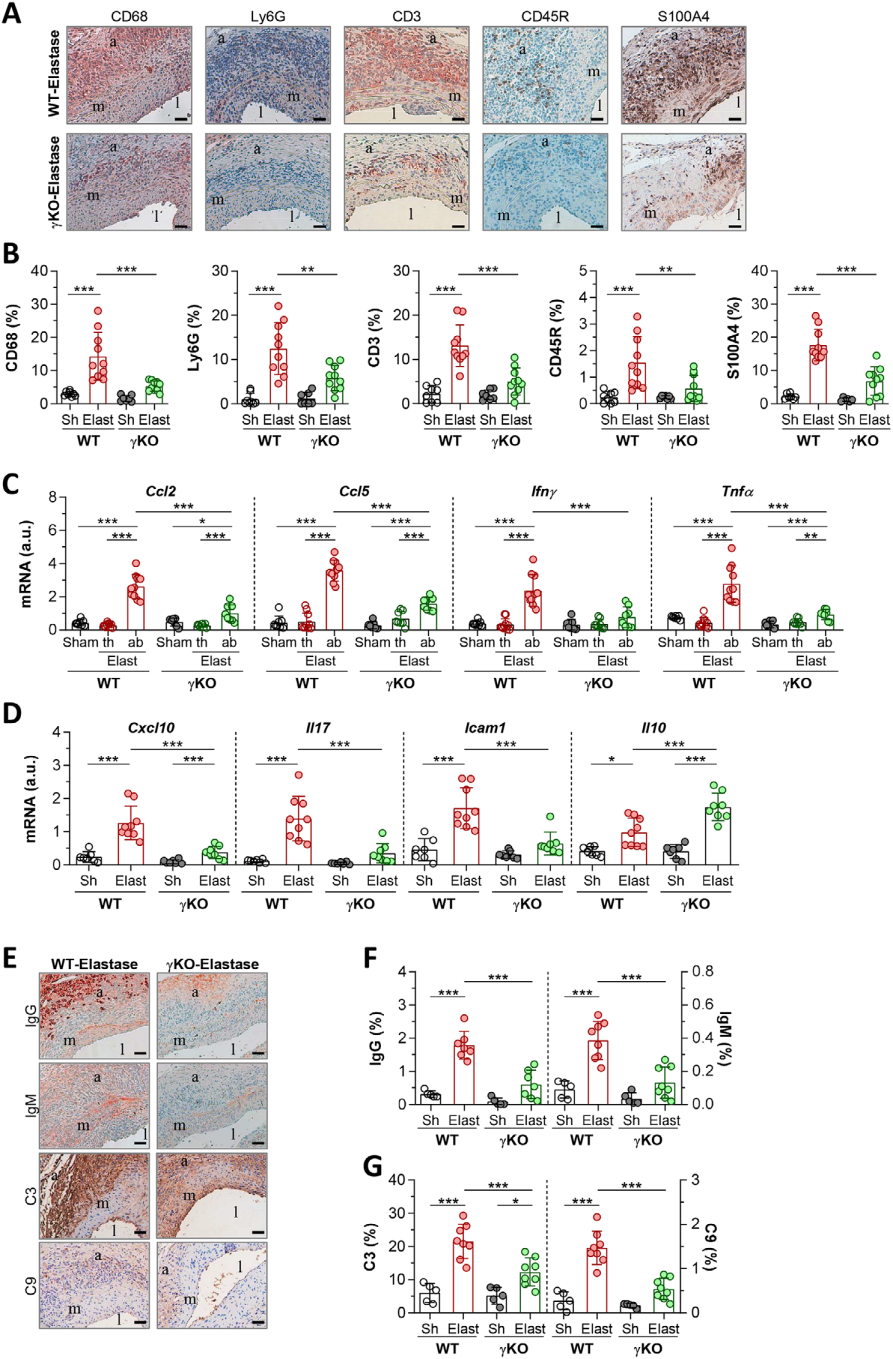
Reduced inflammation and immune response in AAA lesions from FcγR deficient mice. (A) Representative immunohistochemical images (scale bars, 50 μm) of macrophages (CD68), neutrophils (Ly6G), T lymphocytes (CD3), B cells (CD45R) and fibroblasts (S100A4) in abdominal aortic sections from WT and γKO mice after 14 days of elastase perfusion. (B) Quantitative analysis of cell type contents expressed as percentage of positive area in WT (Sham, *n* = 8; Elastase, *n* = 10) and γKO (Sham, *n* = 7; Elastase, *n* = 10) mice. (C) Quantitative real‐time PCR analysis of chemokines and cytokines in the abdominal aorta of saline‐perfused mice (WT‐Sham, *n* = 8; γKO‐Sham, *n* = 7) and in the thoracic (th) and abdominal (ab) aorta of elastase‐perfused mice (WT‐Elastase, *n* = 10; γKO‐Elastase, *n* = 8). (D) Gene expression of pro‐ and anti‐inflammatory genes in abdominal aorta of WT (Sham, *n* = 7; Elastase, *n* = 9) and γKO (Sham, *n* = 7; Elastase, *n* = 8) mice. PCR values normalized to 18S rRNA are expressed as arbitrary units (a.u.). (E) Representative images (scale bar, 50 μm) of antibodies and complement immunostaining in AAA lesions. Quantitative analysis of IgG and IgM (F), C3 and C9 (G) positive areas in WT and γKO mice (Sham, *n* = 5; Elastase, *n* = 8). Results are presented as individual data points and mean ± SD of the mouse groups. *P < 0.05, **P < 0.01, and ***P < 0.001 (one‐way ANOVA plus Bonferroni test). l, lumen; m, media; a, adventitia

To evaluate whether FcγR affects local inflammation, aortic mouse sections were analyzed for different infiltrating cell types. In comparison with WT mice, AAA lesions from γKO mice showed significant decreases in accumulated CD68^+^ macrophages, Ly6G^+^ neutrophils, CD3^+^ T lymphocytes, CD45R^+^ B cells, and S100A4^+^ fibroblasts (Figure 3A, B). Concomitantly, real‐time PCR analysis revealed a decreased expression of proinflammatory chemokines (CCL2 and CCL5) and cytokines (IFNγ and TNFα) in abdominal aorta from γKO mice compared with WT mice, whereas the gene expression levels in thoracic aorta were not altered (Figure 3C). AAA lesions in γKO mice also exhibited lower expression of inflammatory mediators (CXCL10, IL‐17, and ICAM1) and upregulated antiinflammatory cytokine IL‐10 (Figure 3D and Supplementary Figure S3A).

Characterization of immune components revealed a lower content of antibodies IgG and IgM (Figure 3E, F) and complement factors C3 and C9 (Figure 3E, G) in γKO. Moreover, the gene and protein expression levels of activating FcγR (I, III and IV) were decreased in γKO aorta, while the inhibitory FcγRIIB expression remained unaltered (Supplementary Figure S3B‐D).

**FIGURE 4:**
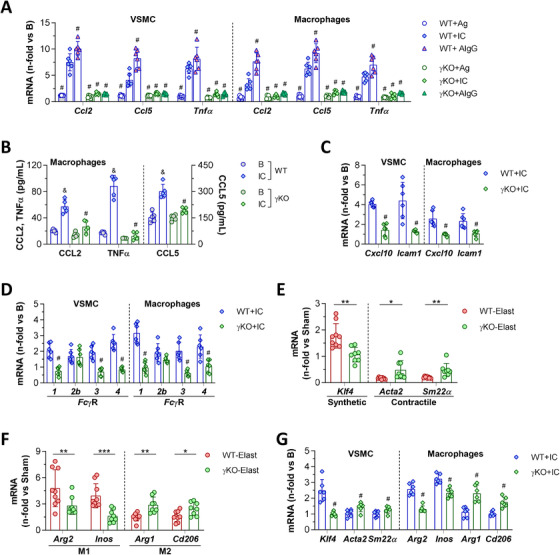
FcγR modulate inflammatory genes and cell polarization markers in vitro and in vivo. (A) Quantitative real‐time PCR analysis of CCL2, CCL5, and TNFα in primary VSMC and BM‐derived macrophages from WT and γKO mice after 24 hours of stimulation with fibrinogen alone (Ag), fibrinogen‐IgG IC or heat‐aggregated mouse IgG (AIgG). (B) ELISA test for protein secretion in conditioned media from BM‐derived macrophages under basal conditions or stimulated with fibrinogen‐IgG IC for 24 hours. IC‐induced mRNA expression of inflammatory genes (C) and activating/inhibitory FcγR (D) in WT and γKO cells. In vivo assessment of the mRNA expression of phenotypic markers for VSMC (E) and macrophages (F) in aortic tissues from elastase‐perfused mice. (G) In vitro effect of IC on the phenotype marker expression in VSMC and macrophages. PCR values normalized by 18S rRNA endogenous control are analysed in duplicate and expressed as fold increases over basal conditions (A, C, D, G) or Sham mice (E, F). Results presented as individual data points and mean ± SD correspond to *n* = 5‐6 independent in vitro experiments and the total animals per group (WT‐Elastase, *n* = 9; γKO‐Elastase, *n* = 8) analyzed in duplicate. *P < 0.05, **P < 0.01, and ***P < 0.001 vs Sham mice; ^&^ *P *< 0.05 vs basal; ^#^ *P *< 0.05 vs WT+IC (Mann‐Whitney test or Kruskal‐Wallis plus Dunn's test)

To further investigate the cell‐specific functions of FcγR, we established an in vitro AAA microenvironment by incubating primary mouse VSMC and BM‐derived macrophages with fibrinogen‐antifibrinogen IgG IC. Fibrinogen was chosen as antigen system based on previous observations of antifibrinogen deposits in aneurysm and inflammatory diseases.^14^, ^34^ We also used heat‐aggregated IgG as an IC mimetic without antigen participation. In VSMC and macrophages from WT mice, the engagement of FcγR by fibrinogen‐IgG IC or IgG aggregates increased the gene expression of CCL2, CCL5, and TNFα; an effect not seen with antigen (fibrinogen) alone (Figure 4A). In both cell types, ELISA revealed high levels of secreted CCL2, CCL5 and TNFα proteins in conditioned media following 24 hours exposure to IC (Figure 4B and Supplementary Figure S4A). We also detected upregulation of CXCL10 and ICAM1 mRNA by IC (Figure 4C), without changes in cell viability (Supplementary Figure S4B, C). By contrast, γKO cells showed an attenuated response to IgG IC stimulation, with significant decreases in inflammatory genes (Figure 4A, C) and cytokine/chemokine secretion (Figure 4B and Supplementary Figure S4A). Moreover, in agreement with in vivo observation, γ‐chain deficiency resulted in the loss of activating FcγR isoforms, while FcγRIIB expression was similar in γKO and WT cells (Figure 4D).

### 3.4 Role of FcγR on vascular cell phenotypes, metalloproteinase activity, and oxidative stress

Because phenotypic and functional switch of vessel cells is an important early step in AAA development,^35^, ^36^ we next examined whether loss of activating FcγR influences VSMC and macrophage phenotypes. Compared with Sham‐operated mice, AAA lesions in WT mice exhibited an increased mRNA expression of KLF4 (synthetic VSMC phenotype marker) and lower levels of ACTA2 and SM22α (contractile VSMC markers) (Figure 4E), alongside upregulated M1 pro‐inflammatory macrophage markers (ARG2 and iNOS) and downregulated M2 reparative genes (ARG1 and CD206) (Figure 4F). By contrast, AAA lesions in γKO mice displayed a decline in synthetic VSMC and M1 markers, and higher expression of contractile VSMC and M2 genes (Figure 4E, F). Calculation of synthetic/contractile VSMC and M1/M2 macrophage ratios confirmed significant differences between WT and γKO mice (VSMC ratio, 4.9±0.4 vs 1.2±0.1; Macrophage ratio, 2.8±0.2 vs 0.9± 0.1; *P *< 0.0001 for both). In cultured VSMC exposed to fibrinogen‐IgG IC, γ‐chain deficiency decreases synthetic marker and increases contractile genes (Figure 4G). Moreover, macrophages from γKO mice displayed a significant reduction of M1 genes and increased M2 markers when compared to IC‐stimulated WT macrophages (Figure 4G), despite both cells exhibiting similar differentiation potential in the presence of conventional M1‐ and M2‐polarizing factors (Supplementary Figure S4D).

**FIGURE 5:**
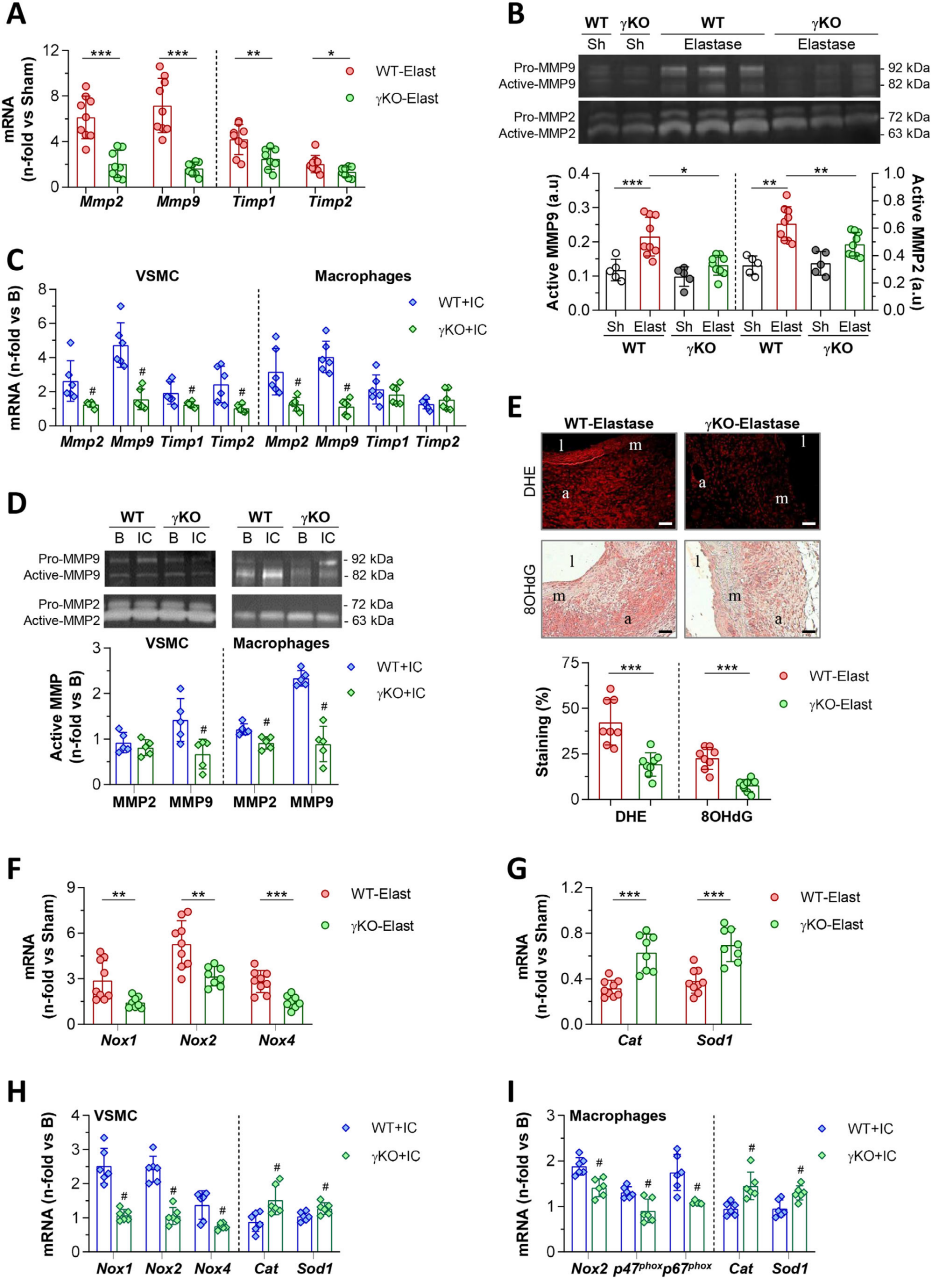
Reduced metalloproteinase activity and oxidative stress by FcγR deficiency. (A) Quantitative real‐time PCR analysis of MMP and TIMP mRNA in abdominal aortic samples from WT‐Elastase (*n* = 9) and γKO‐Elastase (*n* = 8) mice. (B) Gelatin zymography analysis in serum samples from WT and γKO mice (Sham, *n* = 5; Elastase, *n* = 9). Shown are representative gels and densitometric analysis of active MMP2/9 bands expressed as arbitrary units (a.u.). (C) Analysis of MMP and TIMP mRNA expression in VSMC and BM‐derived macrophages from WT and γKO mice after 24 hours of stimulation with fibrinogen‐IgG IC (*n* = 6). (D) Representative zymograms in cell supernatants from primary VSMC and macrophages under basal conditions and 24‐hour following IC stimulation. Densitometric analysis of active MMP2/9 bands relative to basal conditions (*n* = 5). (E) Representative images (scale bars, 50 μm; l, lumen; m, media; a, adventitia) and quantification of superoxide anion (DHE fluorescence) and DNA oxidative stress marker (8OHdG immunoperoxidase) in AAA lesions from WT and γKO mice after 14 days of elastase perfusion (*n* = 8 mice per group). Gene expression analysis of NOX subunits (F) and antioxidant enzymes (G) in abdominal aortic tissues from WT‐Elastase (*n* = 9) and γKO‐Elastase (*n* = 8) mice. Expression of redox balance enzymes in primary VSMC (H) and macrophages (I) stimulated with fibrinogen‐IgG IC for 24 hours (*n* = 6). PCR values normalized by 18S rRNA endogenous control are analysed in duplicate and expressed as fold increases vs Sham mice (A, F, G) or cell basal conditions (C, H, I). Results are presented as individual data points and mean ± SD. *P < 0.05, **P < 0.01, and ***P < 0.001 vs Sham mice; ^#^ *P *< 0.05 vs WT+IC (one‐way ANOVA plus Bonferroni test or Mann‐Whitney test)

Phenotypic switching in AAA lesions from WT mice was accompanied by an increased expression of extracellular matrix degrading enzymes (MMP2 and MMP9) and their inhibitors (TIMP1 and TIMP2) (Figure 5A), along with higher serum gelatinolytic activities (Figure 5B) compared with Sham groups. The excessive expression of MMPs and TIMPs was significantly reduced in γKO mice (Figure 5A). In fact, the MMP/TIMP ratios were lower in γKO compared with WT mice (MMP2/TIMP2, 1.6± 0.3 vs 3.1±0.1, *P *= 0.001; MMP9/TIMP1, 0.7± 0.1 vs 1.9±0.3, *P *= 0.003), suggesting a reduced proteolytic balance that was also confirmed by gelatin zymography (Figure 5B). This effect was also observed in vitro, where γKO cells exhibited lower MMP and TIMP mRNA expression in response to IgG IC (Figure 5C) and attenuated gelatinolytic activity (mainly MMP9) in cell supernatants (Figure 5D) compared with WT cells.

We next evaluated the impact of FcγR deficiency on the oxidative stress response during AAA. Compared with WT mice, AAA lesions in γKO mice exhibited lower levels of superoxide anion (DHE fluorescence) and oxidative DNA damage product (8OHdG immunostaining) (Figure 5E). Real‐time PCR analysis revealed significant reductions of superoxide‐generating enzymes, including phagocytic NOX2 and nonphagocytic NOX1 and NOX4 isozymes, in aortas of elastase‐infused γKO mice (Figure 5F), and higher expression of antioxidant enzymes (Catalase and SOD1) (Figure 5G). Moreover, in vitro formed IC markedly induced the mRNA expression of prooxidant enzymes both in WT VSMC (NOX1, 2, and 4) and WT macrophages (NOX2, p47^phox^, and p64^phox^), whereas antioxidant genes were unmodified. Unlike WT, γKO cells showed reduced expression of NOX isoforms and a slight, but significant increase of Catalase and SOD1 (Figure 5H, I).

### 3.5 Involvement of Syk in FcγR‐mediated responses during AAA formation

**FIGURE 6:**
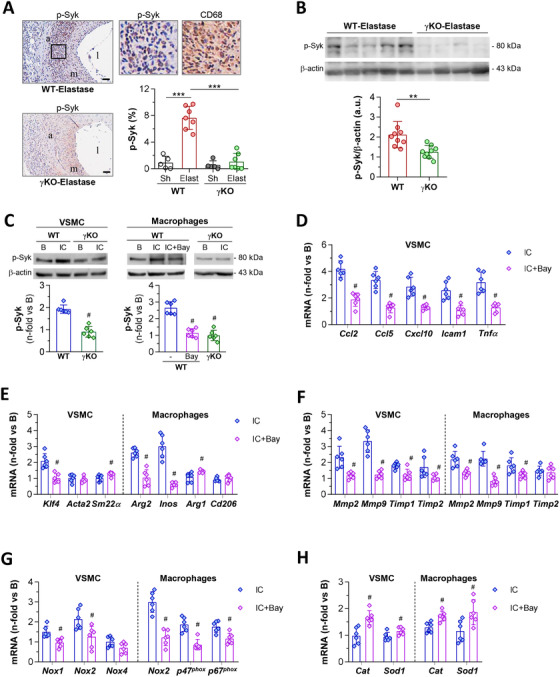
Syk activation mediates FcγR‐mediated responses in experimental AAA and cultured cells. (A) Representative images (scale bars, 50 μm; l, lumen; m, media; a, adventitia) of p‐Syk immunodetection and quantification of positive area in abdominal aortic sections from WT and γKO mice (Sham, *n* = 5; Elastase, *n* = 7). High‐magnification fields (rectangular areas) show colocalization of p‐Syk with CD68 macrophages. (B) Western blot analysis of p‐Syk and β‐actin (loading control) in aortic protein extracts from WT‐Elastase (*n* = 9) and γKO‐Elastase (*n* = 9) mice. Shown are representative images and the summary of normalized quantification expressed in arbitrary units (a.u.). (C) Western blot analysis of p‐Syk in total cell extracts from WT and γKO cells exposed to fibrinogen‐IgG IC for 24 hours with/without Bay 61‐3606 pretreatment (1 μM, 60 minutes). Representative blots and summary of normalized quantification expressed as fold increases vs basal are shown. Quantitative real‐time PCR analysis of inflammatory genes (D), cell phenotypic markers (E), extracellular matrix remodeling genes (F), pro‐oxidant enzymes (G), and antioxidant enzymes (H) in VSMC and BM‐derived macrophages incubated with IC in the absence or presence of 1 μM Bay 61‐3606. Normalized PCR values are expressed as fold changes relative to basal conditions. Results presented as individual data points and mean ± SD correspond to *n* = 6 independent in vitro experiments and the total animals per group. **P < 0.01 and ***P < 0.001 vs Sham mice; ^#^ *P *< 0.05 vs WT+IC (one‐way ANOVA plus Bonferroni test or Mann‐Whitney test)

Because the tyrosine kinase Syk is a critical signaling event downstream of activating FcγR, we next evaluated the contribution of Syk activity in experimental AAA. Immunohistochemistry (Figure 6A) revealed high phosphorylation levels of Syk in AAA lesions from WT mice, showing colocalization with CD68‐stained areas (Figure 6A and Supplementary Figure S5A). Western blot analysis also confirmed Syk activation in AAA tissues (Figure 6B) and in VSMC and macrophages stimulated with IgG IC (Figure 6C). By contrast, loss of activating FcγR in γKO mice attenuated Syk phosphorylation both in vivo and in vitro (Figure 6A‐C).

In another set of experiments, WT cells were pretreated with Bay 61‐3606, a highly selective and potent inhibitor of Syk that does not inhibit other tyrosine kinases (eg, Lyn, Src, Btk, and Itk).^37^ At the doses used (0.1‐5 μM), Bay 61‐3606 did not affect cell viability of VSMC and macrophages (Supplementary Figure S4B, C) but suppressed Syk phosphorylation (Figure 6C) and the expression of FcγR and inflammatory genes (Figure 6D and Supplementary Figure S5B, C). Moreover, Syk inhibition influenced the phenotypic balance of contractile/synthetic VSMC and M1/M2 macrophages, as evidenced by a significant decrease in KLF4, ARG2, and iNOS, and increased levels of SM22α and ARG1 (Figure 6E). At the same time, decreased expression of MMP2/9 and TIMP1/2 (Figure 6F) and lower gelatinolytic activity in cell supernatants were observed (Supplementary Figure S5D). Syk inhibition also restored redox balance in IC‐treated cells by reducing the expression of NOX1/2 isozymes in VSMC and NOX2 complex in macrophages (Figure 6G), while increasing antioxidant genes in both cell types (Figure 6H).

**FIGURE 7:**
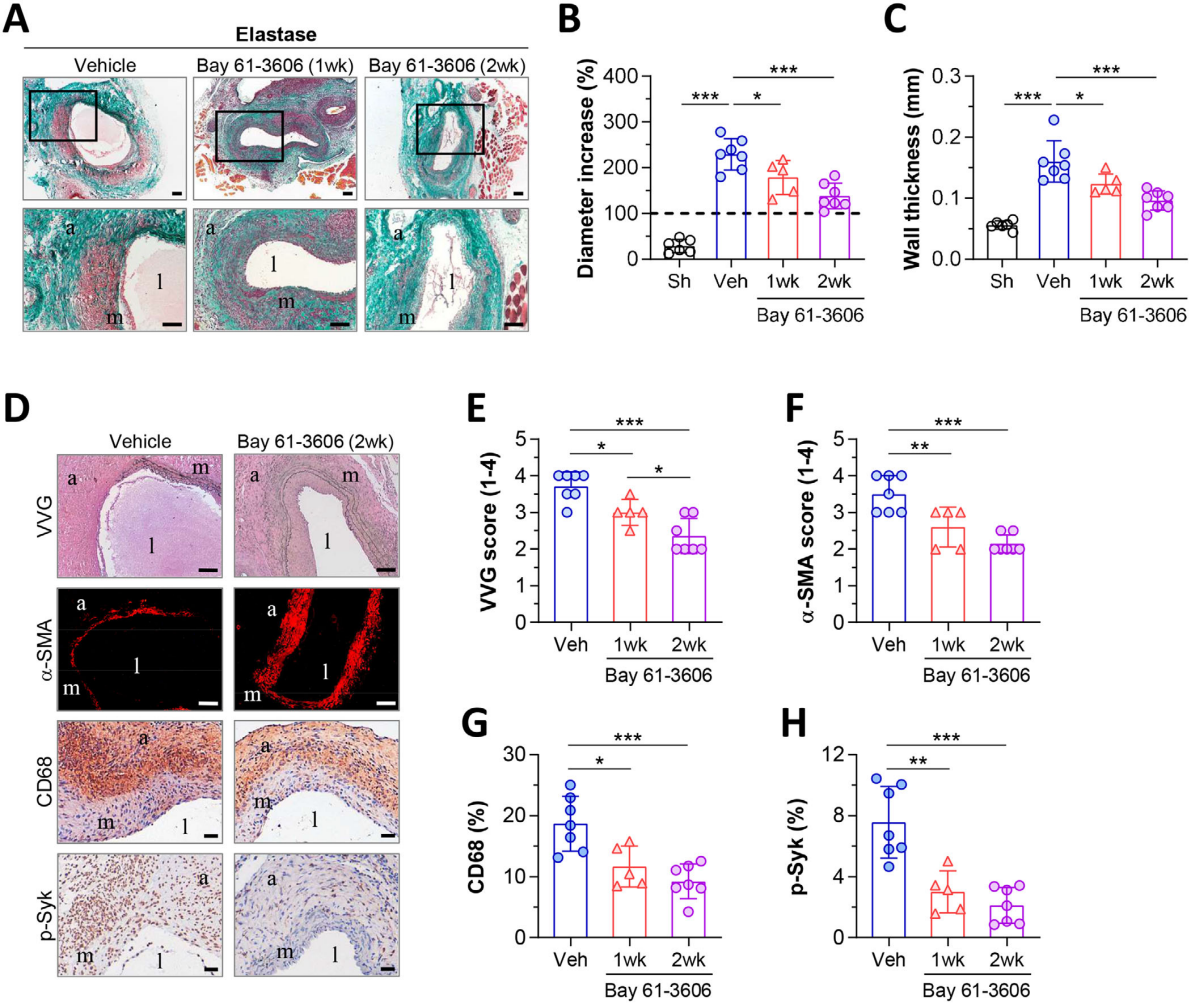
Pharmacological inhibition of Syk attenuates elastase‐induced AAA formation. (A) Representative images (scale bars, 100 μm) and high‐magnification fields (rectangular areas) of Masson's trichrome staining in abdominal aortic sections of elastase‐perfused mice receiving therapeutic Bay 61‐3606 treatment (1 week, from day 7 until day 14), preventive Bay 61‐3606 treatment (2 weeks, from day –1 until day 14) or Vehicle as control. Quantifications of the aortic diameter increase (B) and wall thickness (C) in Masson‐stained sections from saline‐perfused mice (Sham, *n* = 6) and elastase‐perfused groups (Vehicle, *n* = 7; therapeutic 1 week Bay 61‐3606, *n* = 5; preventive 2 weeks Bay 61‐3606, *n* = 7). (D) Representative images of VVG and α‐SMA staining (scale bars, 100 μm), CD68 and p‐Syk immunodetection (scale bars, 50 μm) in Vehicle and Bay 61‐3606 groups (2‐week treatment). (E) Grading of elastin degradation. (F) Grading of medial VSMC. (G) Quantification of CD68^+^ macrophages. (G) Quantification of p‐Syk positive area. Results are presented as individual data points and mean ± SD of the total number of mice per group. *P < 0.05, **P < 0.01, and ***P < 0.001 (one‐way ANOVA plus Bonferroni test or two‐tailed Student's *t*‐test). l, lumen; m, media; a, adventitia

We next analyzed in vivo whether pharmacological inhibition of Syk could play a similar role as γ‐chain deficiency in AAA. To evaluate the effect of Bay 61‐3606 on initiation and progression of AAA, preventive administration therapy was started 1 day before and finished 2 weeks after elastase perfusion. To examine the impact on progression of established AAA, therapeutic Bay 61‐3606 was administered from day 7 and continued 1 week until sacrifice. Compared with Vehicle group, preventive Syk inhibition (2 weeks Bay 61‐3606) reduced aortic expansion, as evidenced by significant decrease of aortic diameter (60% ± 5% vs Vehicle) and wall thickness (60% ± 4% vs Vehicle) (Figure 7A‐C). Therapeutic Syk inhibition (1 week Bay 61‐3606) also limited the progression of existing AAA, although to a lesser extent than preventive therapy (diameter, 78% ± 7%; thickness, 77% ± 5% vs Vehicle) (Figure 7A‐C). AAA lesions from both groups of Bay 61‐3606‐treated mice showed a reduction in elastin degradation, medial VSMC loss, CD68^+^ macrophages and Syk phosphorylation (Figure 7D‐H).

## 4 DISCUSSION

The present study demonstrates the importance of activating FcγR in antibody‐mediated responses during AAA formation: (i) FcγR isoforms are expressed in human and experimental AAA; (ii) blockade of activating FcγR signaling by γ‐chain gene deficiency or Syk kinase inhibition limits AAA development in mice; and (iii) these protective effects associate with changes in chemokines (CCL2/5 and CXCL10), cytokines (TNFα, IFNγ, and IL‐17/10), redox enzymes (NOX1/2/4, catalase, and SOD1) and matrix degrading enzymes (MMP2/9), and phenotypic modulation of VSMC and macrophages.

Autoantibodies specific for self‐derived epitopes participate in vascular inflammation and remodeling through mechanisms including phagocytosis and cell activation via FcγR following IC and complement deposition.^38^ Studies in knockout mice lacking single or multiple FcγR isoforms have improved understanding of IgG‐FcγR interactions in autoimmune and cardiovascular diseases, particularly atherosclerosis.^20^, ^22^, ^23^ In the context of AAA, gene sequencing identified upregulated “B cell receptor signaling” and “Fc receptor‐mediated phagocytosis” pathways in mouse AAA,^39^, ^40^ while microarray analysis^25^ and immunohistochemistry^26^ showed FcγRIIB expression in human AAA. Our study reveals expression of activating, γ‐chain‐associated FcγR (human IA and IIIA; mouse I, III, and IV) and inhibitory ITIM‐bearing FcγRIIB (human/mouse) in the media and adventitia of AAA lesions. Human AAA also express other relevant subtypes including activating ITAM‐bearing FcγRIIA and neutrophil‐specific glycosylphosphatidylinositol‐anchored FcγRIIIB. Noticeably, FcγR colocalized with IgG deposits in human and mouse AAA lesions and were expressed by VSMC and macrophages both in vivo and in cultured cells exposed to IgG IC, thus implicating intrinsic and infiltrating cells in the immune responses during AAA formation.

We demonstrate that IgG‐FcγR‐dependent inflammation in the aortic wall drives AAA formation. Indeed, mice lacking the common γ‐chain of activating FcγRI/III/IV isotypes were protected against elastase‐induced AAA and showed smaller aortic dilation, preserved elastin integrity, and reduced content of macrophages, neutrophils, T and B lymphocytes, and fibroblasts. This was accompanied by a decline in inflammation and oxidative stress in mouse aorta. In line with earlier studies,^20^, ^29^ γ‐chain deficiency limited FcγRI/III/IV expression, but not FcγRIIB, revealing a change from activation to inhibitory state. Thus, it is conceivable that activating FcγRs are required for initiating IC‐mediated inflammation in aortic wall, but modulated by FcγRIIB ability to inhibit effector functions of autoantibodies and immune receptors, as reported in other diseases.^24^, ^41^

The presence of B lymphocytes and Igs in AAA lesions implicates antibody‐mediated humoral immunity.^5^, ^15^ Analysis of IgG from human AAA specimens revealed reactivity to connective tissue components containing fibrinogen‐like motifs.^8^, ^9^ Moreover, plasma levels of IgG against high‐density lipoproteins^11^ and phospholipids^10^ correlate with AAA size, while antibodies to malondialdehyde‐acetaldehyde adducts help distinguish between AAA and atherosclerosis.^12^ In mice, depletion in B cells (and hence antibodies) protects against aneurysm development.^42^, ^43^ Herein, functional deficiency in activating FcγR associates with a reduced content of B cells and antibodies in AAA lesions. We also found lower deposition of complement C3 (activated by classical, alternative and lectin pathways) and C9 (member of membrane attack complex), which are critical mediators of aneurysm.^16^ Zhou et al.^13^ reported that absence of IgG abrogates aortic C3 deposition and protects mice from AAA development. Moreover, IgG antifibrinogen antibodies recognizing specific epitopes in aneurysmal tissues participate in elastase‐induced AAA by activating lectin and alternative complement pathways.^14^ In agreement, we hypothesize that elastase perfusion induces alterations of structural components (eg, proteoglycans, collagens, elastins and glycoproteins) or circulating blood proteins (eg, fibrinogen) in the mouse aortic wall, thus unmasking neoantigens whose recognition by IgG antibodies activates complement and FcγR‐dependent responses to independently and cumulatively promote vascular inflammation.

The aneurysm‐resistant phenotype of γKO mice associated with lower macrophage content and was partially rescued by adoptive transfer of FcγR‐expressing macrophages, thus uncovering the importance of monocytic FcγR in AAA. Monocytes/macrophages actively contribute to initiation and progression of AAA, where local microenvironment promotes polarization toward multidimensional spectrum of phenotypes.^36^ During early stages of mouse AAA, pro‐inflammatory M1 macrophages contribute to oxidative stress, inflammation, and vascular remodeling,^5^ whereas anti‐inflammatory M2 at later phases help prevent expansion and/or rupture.^44^ Cytokine and redox balance also influence the magnitude of monocytic FcγR‐mediated responses.^45^ Accordingly, we observed that γ‐chain deficiency downregulated pro‐inflammatory M1 markers and NOX isozymes, while promoting anti‐inflammatory M2 and antioxidant genes in AAA lesions. Altered balance between MMPs and their inhibitor TIMPs was also found in γKO mice, hampering the MMP2/9 enzymatic activity implicated in AAA expansion and progression towards rupture.^46^ In vitro, macrophage FcγR ligation by fibrinogen IC recapitulates the gene profile of AAA lesions, harmonizing earlier findings with IC containing native and citrullinated fibrinogen.^14^, ^34^ It is reported that FcγRIIIA/IIA engagement associates with M1 polarization,^47^ whereas FcγRIIB promotes M2b immunosuppressive phenotype.^48^ Thus, it is likely that, in the absence of activating FcγR, an FcγRIIB‐mediated efficient removal of IgG IC from the damaged aorta may lower vascular inflammation and oxidative stress, thereby forestalling AAA.

VSMC dysfunction, apoptosis, and plasticity contribute to vascular damage during AAA. Injured VSMC undergo phenotypic transition to a synthetic state characterized by increased migration, proliferation, and extracellular matrix remodeling.^35^ Oxidative stress also modulates VSMC phenotypic switching in aneurysm, as shown by reduced AAA incidence in NOX‐deficient mice^49^ or VSMC‐specific catalase transgenic mice.^50^ Our results in elastase‐infused mice and IC‐stimulated cells indicate VSMC phenotypic switch characterized by downregulation of contractile and antioxidant genes, alongside increased synthetic phenotype markers, proinflammatory cytokines, MMPs and NOX activity, which are all reversed with γ‐chain deficiency. In phagocytes and vascular cells, FcγR engagement by IgG and IC, but also other ligands such as C‐reactive protein (an acute phase protein highly increased in AAA patients)^51^ regulates cytokine release, apoptosis and proliferation.^20^, ^52^ The present study expands on the regulatory role of FcγR on VSMC functions and plasticity and the mechanisms behind the direct immunological effects of VSMC in AAA.

Apart from macrophages and VSMC, distinct FcγR‐bearing effector cells such as neutrophils and fibroblast may contribute to mouse AAA formation. Neutrophils, early responders to acute inflammation during vessel wall injury are quickly recruited in experimental AAA and found in the intraluminal thrombus of human AAA.^5^ Resting neutrophils express FcγRIIA/IIIB involved in phagocytosis and removal of soluble IC within the vasculature, while inducible FcγRI triggers neutrophil antibody‐dependent cytotoxicity.^17^ Vascular injury also promotes phenotypic switch of adventitial fibroblasts into migratory myofibroblasts, which modify redox state and matrix composition and accelerate macrophage recruitment and aortic dilation.^53^ It is reported that activating FcγR mediate differentiation of cardiac fibroblast precursor cells in a cardiomyopathy model.^54^ Future efforts are needed to understand the antibody‐mediated mechanisms of these cells in AAA disease.

Mechanistically, our study reveals that molecular events downstream vascular FcγR activation in AAA depend on Syk phosphorylation. In leukocytes, FcγR crosslinking induces tyrosine phosphorylation ITAM‐bearing γ‐chain by Src kinases and Syk recruitment to further stimulate multiple kinases and transcription factors.^19^ Syk expression/phosphorylation is abnormally increased in immune and cardiovascular diseases.^55^ Our study showing Syk activation in elastase mouse model confirms previous findings in human AAA^27^ and also identifies an FcγR‐Syk‐dependent pathway in macrophages and VSMC. Indeed, preventive rather than therapeutic treatment with Syk inhibitor limited AAA formation to the same degree as γ‐chain deficiency, and reduced inflammation, oxidative response, and phenotype switching in VSMC and macrophages. This is consistent with Syk‐mediated inhibition of cytokine and MMP9 secretion in human AAA tissue cultures,^27^ M1 phenotype, and VSMC proliferation/migration.^37^, ^56^

In this study, we cannot discard that γ‐chain and Syk may be coupled to transducing systems other than FcγR. In this way, associated γ‐chain to IgA and IgE receptors, T cell/CD3 receptor complex and glycoprotein VI regulate leukocyte and platelet activation,^17^, ^19^ while Syk kinase couples other ITAM‐bearing immune receptors (eg, B‐ and T‐cell antigen receptors and C‐type lectin receptors).^57^ Recent experiments propose a direct role of IgE Fc receptor in activation of T cells, mast cells and macrophages in angiotensin II‐induced AAA,^7^ while B cell and Ig deficiency suppressed Syk activation and protects mice from CaCl_2_‐induced AAA.^43^ Facing the multiple combinations of γ‐chain and Syk, the occurrence of an interaction by a mechanism similar to those previously proposed cannot be excluded in our AAA model.

## 5 CONCLUSION

Our work demonstrates a pathogenic role of IgG IC interaction with activating FcγR present in infiltrating macrophages and VSMC during AAA formation. Genetic and pharmacological inhibition of FcγR‐dependent activation limits experimental AAA through coordinated regulation of inflammation, oxidative stress, proteolytic activity, and phenotype transition. Therefore, therapeutic modulation of FcγR balance and/or downstream molecules may be an attractive target to downregulate vascular immunoinflammatory damage in AAA patients. This approach could also benefit atypical inflammatory variants of AAA associated to infections (eg, Chlamydia pneumoniae, cytomegalovirus, or SARS‐CoV‐2)^58^ and systemic inflammatory diseases (eg, IgG4‐related disease),^59^ as well as immunocompromised patients at high cardiovascular risk.^60^ In fact, blockade of activating FcγR and/or enhanced inhibitory FcγRIIB are underlying mechanisms of intravenous Igs currently used as first‐line or adjunct therapy in autoimmune and immunodeficient diseases^61^; vasculoprotective effects, including aortic aneurysm reduction, has been reported in many cases (eg, Kawasaki disease and systemic lupus erythematosus).^62^ Future studies determining the aortic expression profile of FcγR in these patients are warranted.

## Supporting information

Supporting information
