# Supplementary material for: Fcγ receptor activation mediates vascular inflammation and abdominal aortic aneurysm development
Source: Clin Transl Med. 2021 Jul 4;11(7):e463. doi: 10.1002/ctm2.463 (PMC8255062; doi:10.1002/ctm2.463)

Manuscript ID CTM2-2021-02-0352-R1

Fcγ receptor activation mediates vascular inflammation and abdominal aortic aneurysm development.

Lopez-Sanz L, et al.

SUPPLEMENTARY FIGURE LEGENDS

Supplementary Figure S1: Human and mouse FcγR system. (**A**) Structure diagram of IgG receptors at the cell membrane and their association or not to the γ-chain dimer. The mouse FcγR family contains orthologous receptors for human FcγRIA (mouse FcγRI), FcγRIIA (mouse FcγRIII), FcγRIIIA (mouse FcγRIV) and FcγRIIB (mouse FcγRIIB). Human FcγRIIC and FcγIIIB do not exist in mice. Receptors are labelled as "activating" or "inhibitory", and their relative affinity to IgG is indicated. ITAM, immunoreceptor tyrosine-based activation motif; ITIM, immunoreceptor tyrosine-based inhibitory motif; GPI, glycosylphosphatidylinositol. (**B**-**E**) Colocalization of FcγR with macrophages and VSMC in human AAA samples. Representative double immunofluorescence staining images (scale bar 10μm) showing the expression of FcγRIA (green, **B** and **C**) in $CD68^+$ macrophages (red, **B**) and α-SMA^+ VSMC (red, **C**), and the expression of FcγRIIIA (green, **D** and **E**) in $CD68^+$ macrophages (red, **D**) and α-SMA^+ VSMC (red, **E**). DAPI (blue) stained nuclei. Arrows indicate co-localization (yellow).

Supplementary Figure S2: Adoptive transfer of WT monocytic cells reverses the aneurysm-resistant phenotype of γKO mice. (**A**) Scheme of the adoptive transfer of BM-derived macrophages protocol in the elastase-perfusion AAA mouse model. (**B-D**) Tracking of 5-chloromethylfluorescein diacetate (CMFDA)-labelled donor macrophages transferred into recipient mice on day 7 after elastase perfusion. (**B**) *Ex vivo* imaging system in mouse aorta at 24-48 hours postinjection. (**C**) Representative fluorescence images showing localization of donor macrophages (green) in aortic cross-sections of recipient mouse after 24 hours of adoptive transfer (green-CMFDA, blue-DAPI staining). (**D**) Representative flow cytometry showing the enrichment of macrophages $CD11b^+$ in peripheral blood from recipient mouse after 24 hours of CMFDA-macrophages infusion compared to a control mouse without cell transfer. Bar chart shows the individual percentages of $CD11b^+$ macrophages from 5 adoptive transfer recipients. (**E**) Gross morphological view of abdominal aorta in the adoptive transfer model on day 14. (**F**) Representative images (scale bars, 100μm) and high-magnification fields (rectangular areas) of Masson's trichrome and VVG staining in abdominal aortic sections of the adoptive transfer model of elastase-perfusion AAA. l, lumen; m, media; a, adventitia. Quantification of the aortic diameter increase (**G**) and wall thickness (**H**) in Masson-stained sections. (**I**) Quantification of the elastin degradation score after VGG staining. Results are presented as individual data points and mean±SD of n=5 mice per group. *$p<0.05$, **$p<0.01$ and ***$p<0.001$ (one-way ANOVA plus Bonferroni test).

Supplementary Figure S3: Lack of activating FcγR attenuates immunoinflammatory mediators in AAA lesions. (**A**) Immunodetection of adhesion molecule ICAM1 in abdominal aortic sections after 14 days of elastase perfusion. Representative images (scale bars, 50μm) and quantitative analysis of positive area in WT and γKO mice (Sham, n=5; Elastase, n=7). (**B**) Quantitative real-time PCR analysis of FcγR in abdominal aorta from WT-Elastase (n=8) and γKO-Elastase (n=8) mice. Values normalized by 18S rRNA endogenous control are analysed in duplicate and expressed as fold increases vs Sham mice. (**C**) Representative images (scale bars, 50μm) of FcγR immunohistochemistry in abdominal aortic sections from WT-Elastase (n=8) and γKO-Elastase (n=8) mice. (**D**) Quantitative analysis of positive staining expressed as fold changes vs respective Sham groups. Results are presented as individual data points and mean±SD of the total number of animals per group. *$P<0.05$, **$P<0.01$ and ***$P<0.001$ vs Sham mice (one-way ANOVA plus Bonferroni test or Mann-Whitney test). l, lumen; m, media; a, adventitia.

Supplementary Figure S4: Analysis of protein secretion, cell viability and cell polarization *in vitro*. (**A**) ELISA analysis of CCL2, TNFα and CCL5 protein secretion in conditioned media from VSMC under basal conditions or stimulated with fibrinogen-IgG IC for 24 hours (n=5). (**B-C**) Cell viability assay in mouse VSMC

(**B**) and BM-derived macrophages (**C**) after 24 hours of incubation with the indicated concentrations of Bay 61-3606 in the presence or absence of fibrinogen-IgG IC. Positive control (10% FBS) and negative control (10% DMSO) were used. Cell viability is expressed as percentage vs basal conditions (n=6). (**D**) Phenotype marker expression in BM-marrow macrophages maintained in control conditions (M0) and stimulated for 24 hours with 100 ng/mL of LPS plus 20ng/mL of IFNγ (M1 polarization) and 20 ng/mL of IL-4 (M2 polarization). PCR values normalized by 18S rRNA endogenous control are analysed in duplicate and expressed as fold increases over M0. Results presented as individual data points and mean±SD. $^{\&}P<0.05$ vs basal; $^{\#}P<0.05$ vs WT+IC; $^{\$}P<0.05$ vs M0→M1 polarization (one-way ANOVA plus Bonferroni test or Mann-Whitney test).

Supplementary Figure S5: Involvement of Syk in FcγR-mediated responses. (**A**) Representative confocal fluorescence images for p-Syk (green), CD68 macrophage marker (red) and cell nuclei (blue) staining in mouse AAA lesions. (**B**-**C**) Quantitative real-time PCR analysis of activating/inhibitory FcγR (**B**) and inflammatory genes (**C**) in VSMC and/or BM-derived macrophages treated with Bay 61-3606 (1μM) before stimulation with fibrinogen-IgG IC. Normalized PCR values are expressed as fold changes relative to basal conditions. (**D**) Gelatin zymography assay in cell supernatants from VSMC and BM-derived macrophages. Shown are representative gels and densitometric analysis of active MMP2/9 bands relative to basal conditions. Results presented as individual data points and mean±SD correspond to n=5-6 independent *in vitro* experiments and the total animals per group. $^{**}P<0.01$ vs WT-Elastase mice; $^{\#}P<0.05$ vs WT+IC (Mann-Whitney test).

Lopez-Sanz et al. Supplementary Figure S1

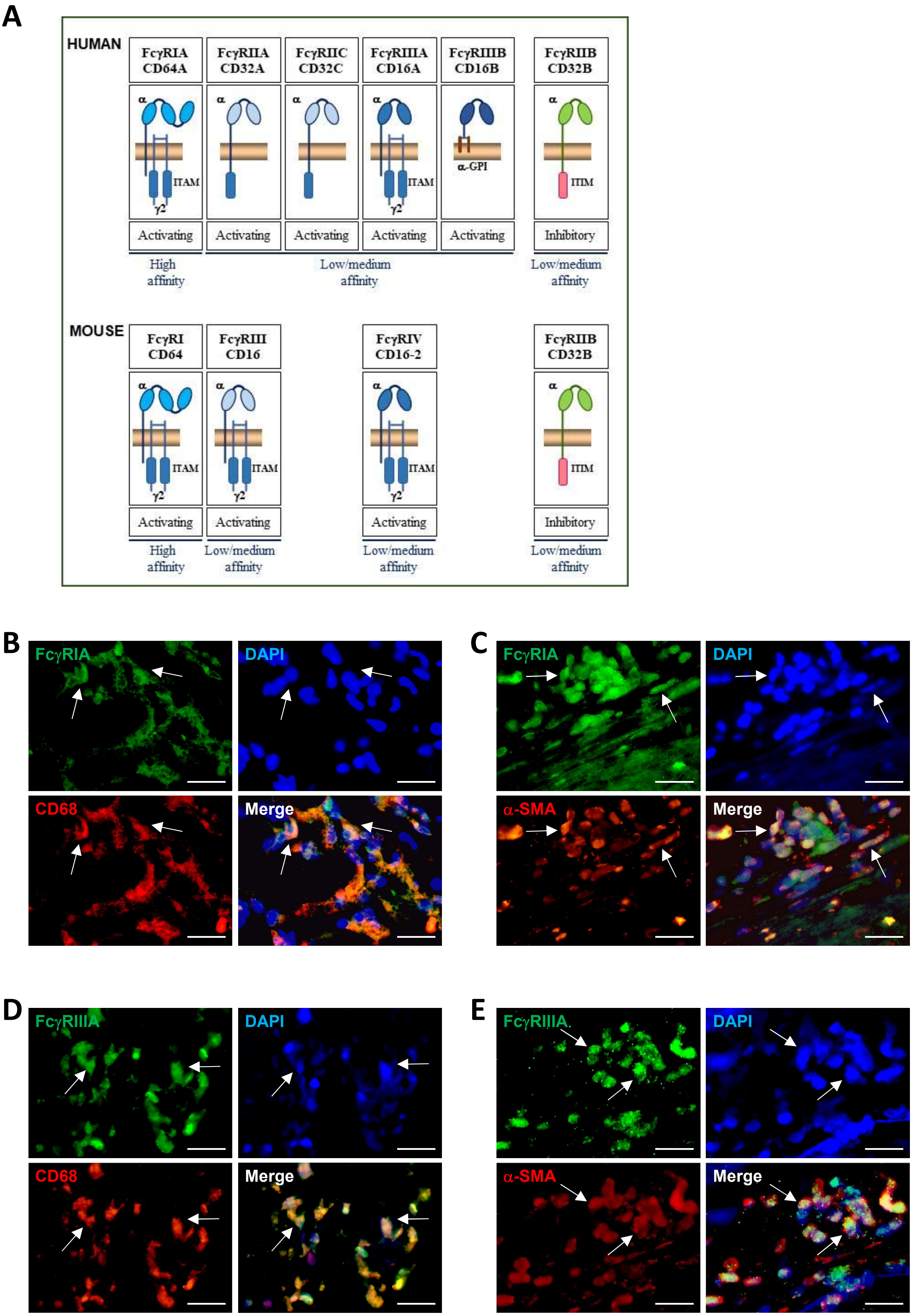

Lopez-Sanz et al. Supplementary Figure S2

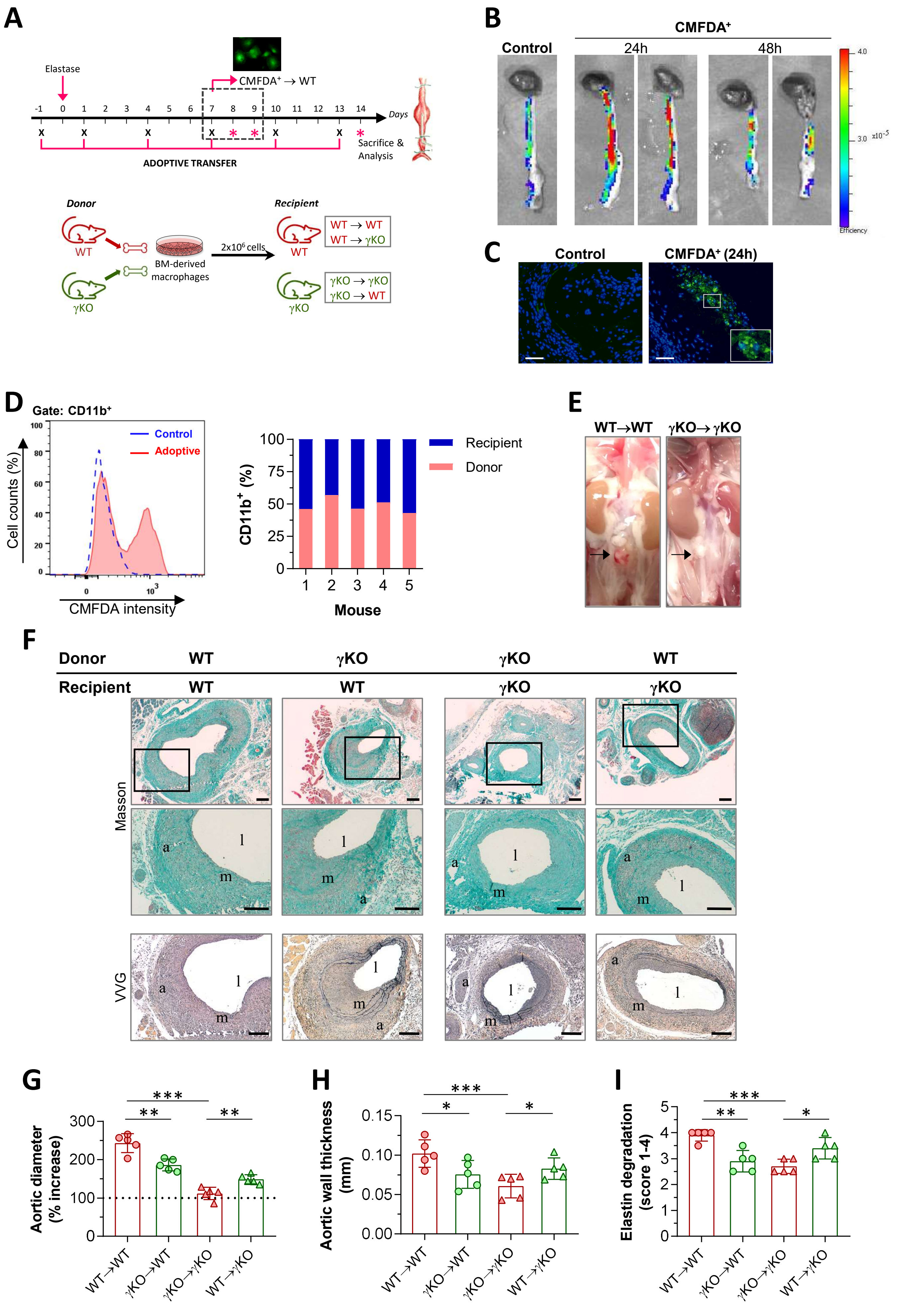

Lopez-Sanz et al. Supplementary Figure S3

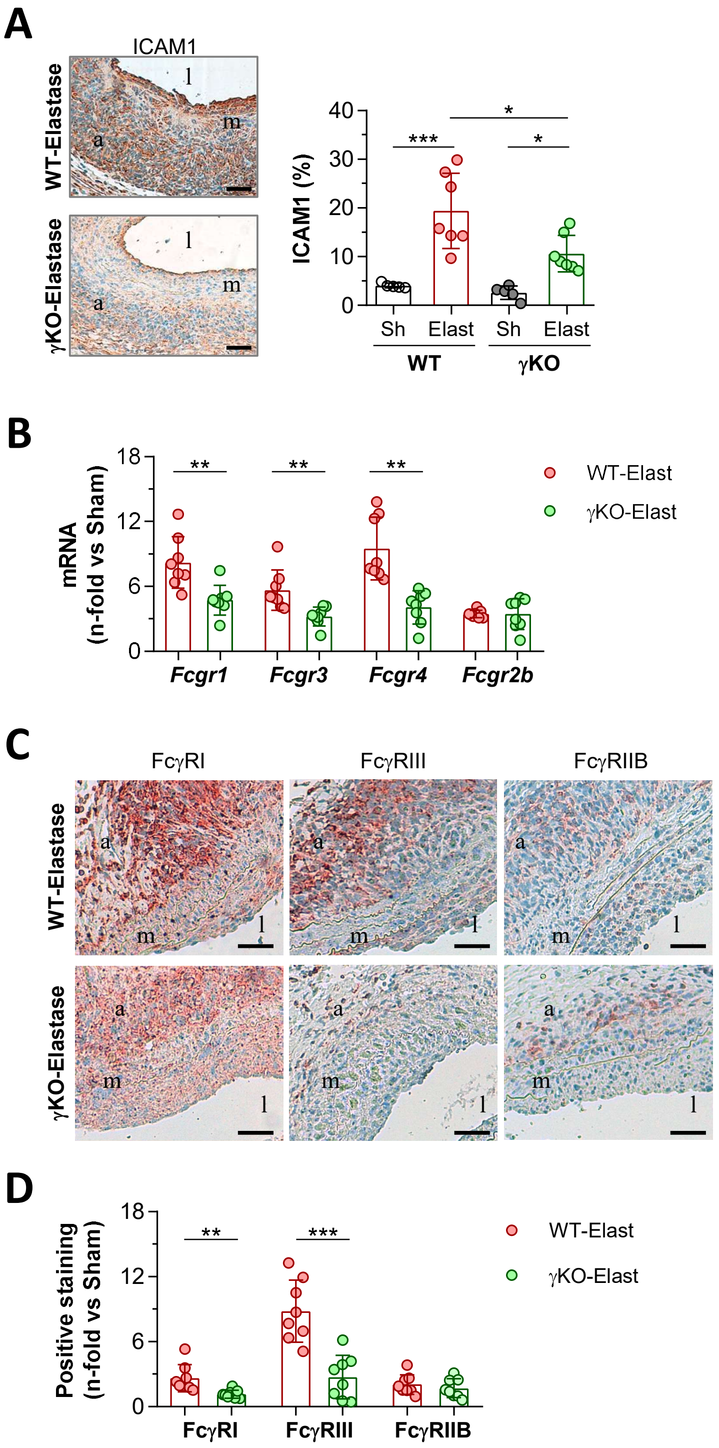

Lopez-Sanz et al. Supplementary Figure S4

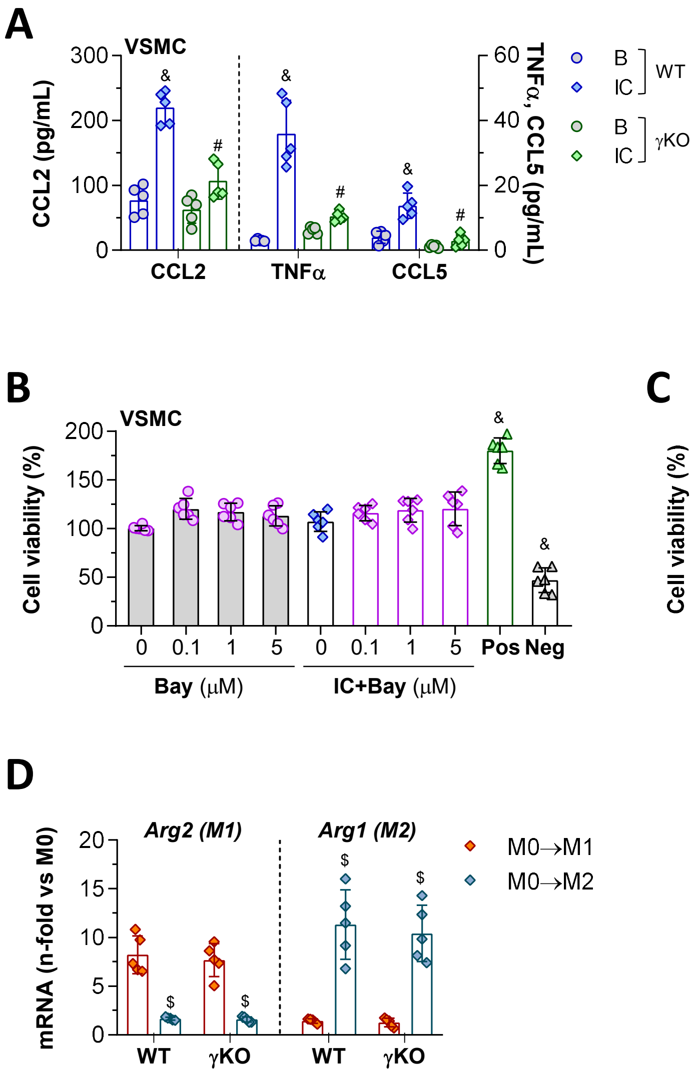

Lopez-Sanz et al. Supplementary Figure S5

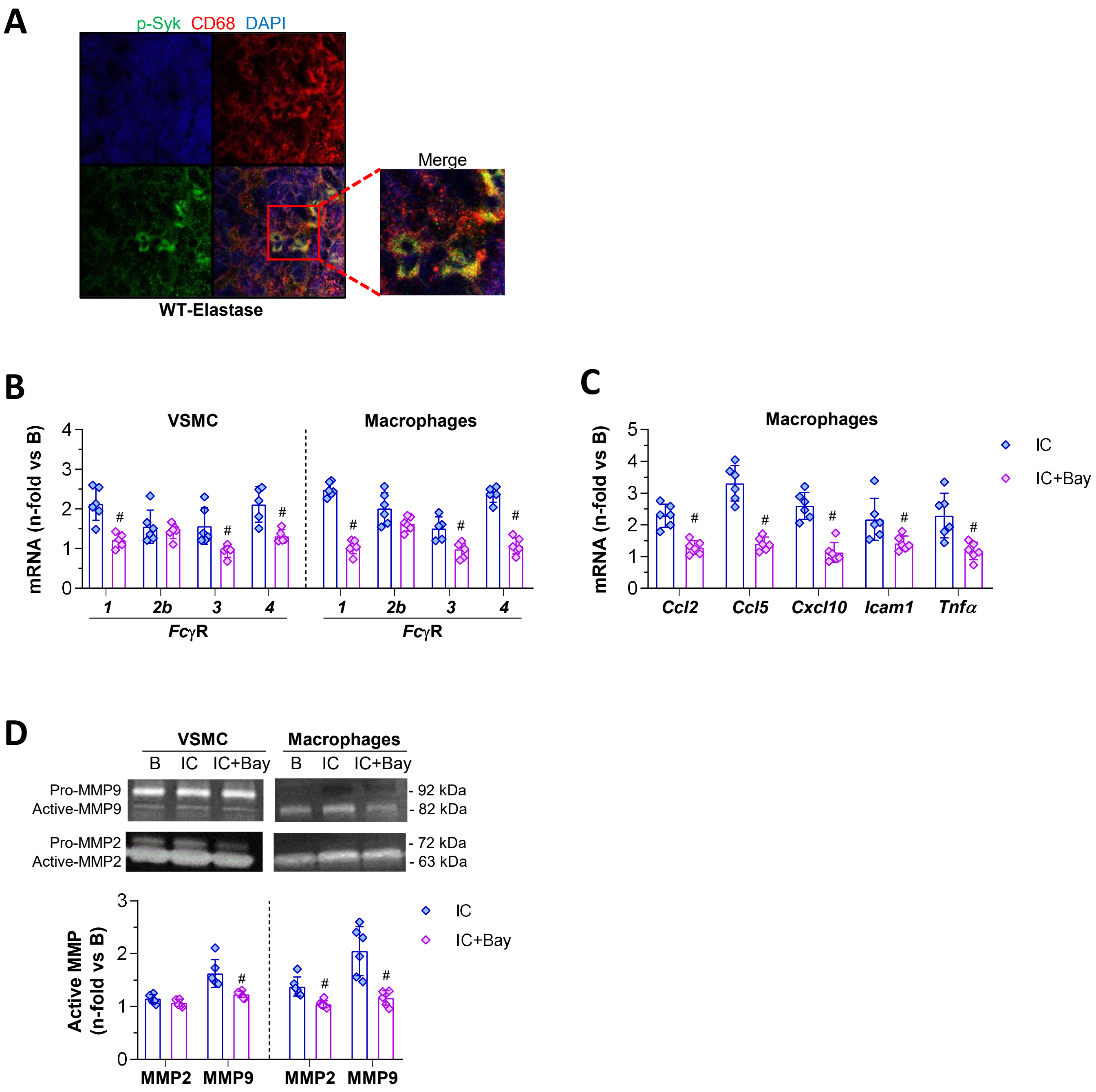

Supplement: Supplementary file 1 — Supporting information [file CTM2-11-e463-s001.pdf]
